# Vesicoureteral Reflux Detected with ^99m^Tc-DTPA Renal Scintigraphy during Evaluation of Renal Function

**DOI:** 10.3889/oamjms.2016.012

**Published:** 2015-12-31

**Authors:** Nevena Manevska, Sinisa Stojanoski, Venjamin Majstorov, Daniela Pop-Gjorcheva, Nikolina Zdraveska, Dafina Kuzmanovska

**Affiliations:** 1 *Institute of Patophysiology and Nuclear Medicine, Faculty of Medicine, Ss Cyril and Methodius University of Skopje, Skopje, Republic of Macedonia*; 2 *University Children’s Hospital, Faculty of Medicine, Ss Cyril and Methodius University of Skopje, Skopje, Republic of Macedonia*

**Keywords:** VUR, renal function, ^99m^Tc-DTPA scintigraphy, ^99m^Tc-DMSA scintigraphy, hydronephrosis

## Abstract

**BACKGROUND:**

Radionuclide techniques, as direct radionuclide cystography and ^99m^Tc-DMSA scintigraphy, have been used in evaluation of vesicoureteral reflux (VUR) and reflux nephropathy (RN) in children. Dynamic ^99m^Tc-DTPA scintigraphy is reserved for evaluation of differential renal function and obstruction in children, where hydronephrosis is detected by ultrasonography (US) pre- or postnatally.

**CASE REPORT:**

Six year old boy was prenatally diagnosed with bilateral hydronephrosis. Postnatal, severe bilateral VUR was detected by voiding urethrocytography. US and ^99m^Tc-DTPA scintigraphy performed in the first month of life showed small left kidney that participated with 2% in the global renal function. Bilateral cutaneous ureterostomy has been performed in order to obtain good renal drainage and promote optimal renal growth. Twelve months later, classic antireflux procedure was done. Control ^99m^Tc-DTPA scintigraphy, 5 ys after antireflux surgery, revealed persisting radioactivity during the diuretic phase, in the left kidney that indicated antireflux procedure failure with VUR reappearance.

**CONCLUSION:**

^99m^Tc-DTPA scintigraphy is the first method of choice for long-term monitoring of individual kidney function in children with VUR and other congenital urinary tract anomalies. Additionally, it can be used as indirect radionuclide cystography when rising of radioactivity in the kidney region, during the diuretic phase can indicate presence of VUR.

## Introduction

Vesicoureteral reflux (VUR) is a congenital defect of the urinary tract leading to retrograde urine flow – from the bladder towards the kidney. It occurs in 1% of the general population and is one of the main risk factors in children for renal scar development after infection of the urinary tract [1].

Several studies stress the importance of diagnosing VUR as a risk factor for repeated urinary tract infections (UTIs), which if left untreated, can lead to serious kidney damage in the future. Especially severe VUR (grades 4 and 5) has been associated with renal damage and represents an important cause of chronic renal failure in children [2].

Radionuclide techniques, as direct radionuclide cystography and cortical scintigraphy with ^99m^Tc-dimercaptosuccinic acid (^99m^Tc-DMSA), have been used in evaluation of VUR and reflux nephropathy in children. The dynamic renal scintigraphy with ^99m^Tc-diethylene triamine pentaacetic acid (^99m^Tc-DTPA) provides important functional information of kidney function in children with VUR.

We aimed to report vesicoureteral reflux detected with ^99m^Tc-DTPA renal scintigraphy during evaluation of renal function in a boy who was prenatally diagnosed with bilateral hydronephrosis.

## Case Report

We present a case of VUR detection during evaluation of renal function by dynamic scintigraphy with ^99m^Tc-DTPA.

**Figure 1:**
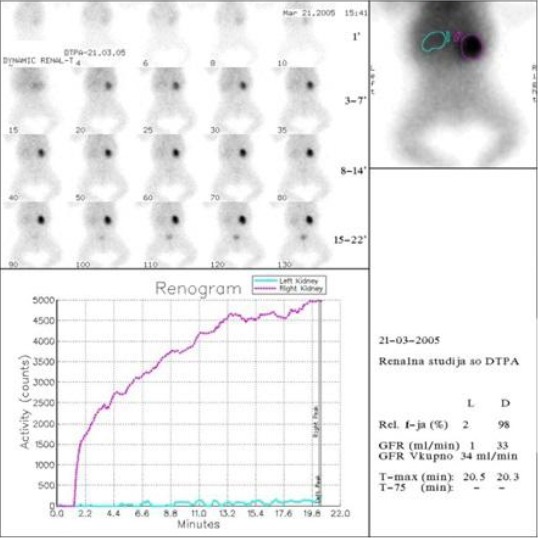
*Dynamic renal scintigraphy with ^99m^Tc-DTPA*.

A 6-year old boy was prenatally diagnosed with bilateral hydronephrosis. Postnatal imaging by voiding urethrocystography revealed severe bilateral VUR (grade IV/V on left and grade III/V on the right side). Renal ultrasonography showed bilateral hydronephrosis and reduction of the renal parenchyma of the left kidney. The renal function was evaluated by dynamic scintigraphy (year 2005). After i.v bolus injection of ^99m^Tc-DTPA, 120 short time frames (4 s in vascular phase and 10 s in dynamic phase) within 20 minutes were taken, matrix size 64 x 64, using one headed SOPHA gamma camera. It has been shown that left kidney participated in the global renal function with only 2% (Fig. 1).

**Figure 2:**
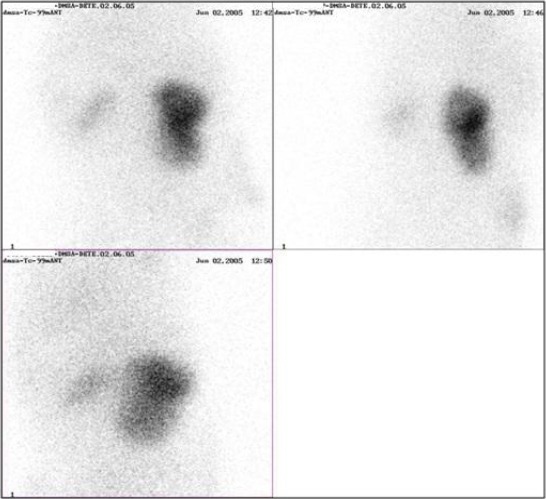
*Renal scintigraphy with ^99m^Tc-DMSA*.

Static ^99m^Tc-DMSA scintigraphy (year 2005) was performed for evaluation of renal cortical morphology. Three hours after the injection of ^99m^Tc-DMSA (dose of 2 MBq/kg body weight), static images in supine position were obtained (posterior-PA, anterior-AP, left oblique-LO and right oblique-RO), using a double-head MEDISO gamma camera, 256 x 256 matrix size (100-300 Kcnts for each image). The scan data showed focal cortical parenchyma defect in the mid-third of the right kidney and very small functional renal tissue on the side of the left kidney - state D according Smelly classification for reflux nephropathy (Fig. 2).

Surgical treatment was implemented, initially bilateral cutaneous ureterostomy have been created in order to obtain good renal drainage and promote optimal renal growth. Classic antireflux procedure (Leadbetter-Politano ureterocystoneostomy) was performed after 12 months.

During the following five years, the child has been regularly checked in outpatient pediatric Nephrology Clinic. Renal functional studies, as degradation products and clearance of endogenous creatinine, were in normal range, as well as proteinuria. The child had neither urinary infection, nor hypertension.

**Figure 3:**
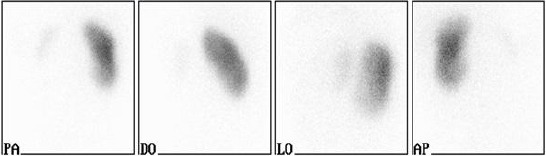
*Renal scintigraphy with ^99m^Tc-DMSA. Relative uptake of ^99m^Tc-DMSA: L = 3%, D = 97%*.

The follow up ^99m^Tc-DMSA scintigraphy (year 2008), showed improvement of the findings at the side of the right kidney, without any change in the left renal function (Fig. 3).

**Figure 4:**
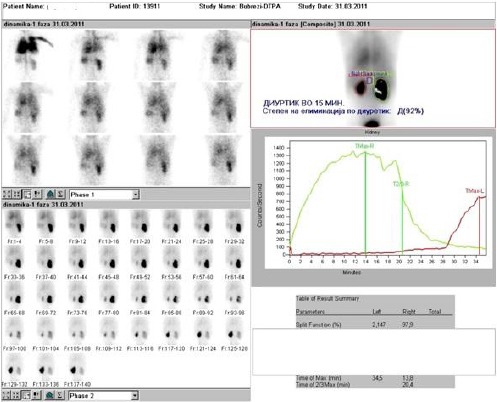
*Dynamic renal scintigraphy with ^99m^Tc-DTPA*.

Three years after (year 2011), control renal diuretic scintigraphy with ^99m^Tc-DTPA was performed. Diuretic (furosemid) was given 15 minutes after the start of the dynamic renal scintigraphy. This scan data showed almost identical findings concerning the renal function in comparison with initial scan. However, during the diuretic phase an increase in the radioactivity in the region of the left kidney was noticed. This finding suggested failure of the antireflux procedure on the left side, with reappearance of VUR (Fig. 4).

## Discussion

Neonatal hydronephrosis (identified if pelvic diameter is > 4mm on antenatal ultrasound) is a common abnormality that can be diagnosed ante- or postnatal, with incidence of 2-9 per 1000 infants [3]. Involving of ultrasonography as a routine test during pregnancy, allows detection of eventually intrauterine anomalies especially if they are performed during 18-20 week of gestation [4].

Among the urinary tract anomalies, hydronephrosis is the most common one. The appearance of intrauterine hydronephrosis was first described by Garett et al., in 1975 [5]. The causative factors of antenatal hydronephrosis (AH) can be categorized into those leading to obstruction (ureteropelvic junction obstruction - UPJO), those leading to reflux (vesicoureteral reflux - VUR) and a group of non-obstructing and non-refluxing “idiopathic” hydronephrosis [6]. VUR as a common cause of AH occurs in 10-15% of them [7].

Primary VUR is associated with congenital defect in the valve mechanism that prevents urine to flow backward from the bladder into the ureters. The valve does not close properly resulting in an ureterovesical junction anomaly. It is diagnosed in the early child age, as a consequence of often repeated UTIs resistant to conservative treatment. Secondary VUR, where valve mechanism is intact, is due to urinary tract malfunction, often caused by infection or increased vesicular pressures associated with obstruction. This conditions result in elevation of the bladder pressure, which distorts the ureterovesical junction [8].

In 1981, international grading system consisting of five grades was established.

The most important consequence of VUR is reflux nephropathy and renal scarring which occurs in 25% of children and younger adults with chronic renal failure. In the study of Ajdinovic et al., children with UTI and VUR (53%) had significantly higher percent of abnormal DMSA findings, than children with UTI without VUR (15%) [9].

VUR is relatively common disorder in childhood which is associated with recurrent urinary tract infection, hydronephrosis, hypertension, renal dysplasia and parenchyma damage, failure to thrive and end-stage renal disease.

Dilating reflux (grades III-V) has been shown to be significantly associated with reflux nephropathy. Prognosis is worst when RN is bilateral. Unilateral RN is compensated for the hypertrophy of the contralateral normal kidney [10].

In the study of Ali et al. the most common causes of hydronephrosis in fetuses are VUR (40.2%), UPJO (32,8%), PUVs (13.4%) and transient hydronephrosis [3]. In the study of Hamid this percent was less for the VUR (17.8), for UPJO (43.6%) and for transient hydronephrosis (20.3%). VUR was identified in 60 (29.7%) of newborns, 48 males and 12 females. The rate of VUR was 27.1% in unilateral hydronephrosis and 34.8% in bilateral.

Farhat reported 48% of neonates with AH had high grade of VUR (IV-V), while Ismail showed 36% of 43 infants with primary VUR had high grade VUR [11, 12].

Direct radionuclide cystography with ^99m^ Tc-labeled agent (sulfur colloid, DTPA, or pertechnetate) is a well-accepted alternative to fluoroscopic VCUG. It is an investigation for initial diagnosis, follow-up examination of children with VUR or for postoperative evaluation after ureteral reimplantation. This technique requires bladder catheterization and it cannot delineate anatomy of bladder and urethra. The advantages of this method include continuous monitoring and imaging, high sensitivity, and a decreased radiation dose for a voiding imaging study [13].

In the study of Thakral et al., DRCG was found to be a sensitive technique for the detection of VUR. There was a direct relationship between the grade of reflux and renal scarring.The study also reveals that there is a cause-and-effect relationship between UTI and renal scarring that is made worse by VUR [14].

The detection of VUR during dynamic renography is unusual. Conventional ^99m^Tc-MAG3 scintigraphy can work as indirect cystography to detect reflux. Nizar et al. evaluated 5 year old boy with moderate bilateral hydronephrosis, with ureteric dilatation presented on US, but VCUG did not demonstrate any VUR and showed normal urethral caliber. Dynamic renal scan with MAG3 and post-void images with calculation of residual urine volume detected the VUR [15].

Arena et al. found VUR in 17% infants with antenatal hydronephrosis. Low grade VUR (I-III) may be missed on antenatal evaluation as there may not be dilatation of the renal pelvis [16].

In our case during control dynamic renal scintigraphy with ^99m^Tc-DTPA, as a standard procedure of evaluation of the renal function in child with surgically treated VUR and reflux nephropathy, progressive accumulation of radioactivity was noted on the late scan images, in the region of the left non-functional kidney. This finding suggested presence of VUR and has been confirmed by subsequent conventional voiding uretrocystography. Consequently, ^99m^Tc-DTPA dynamic renal scyntigaphy in our case served as an indirect radionuclide cystography, giving additional information and helping clinicians to optimize further clinical workout.

In conclusion, early undiagnosed VUR could be a reason for developing urinary tract infection, which can lead to serious renal damage as increasing risk of pyelonephritis and progressive renal failure. The gold standard method for diagnosing VUR is voiding cystourethrography (VCUG), but it is invasive, exposes the patient to radiation and increases the risk of reinfection.

^99m^Tc-DMSA is a sensitive and specific method for detecting renal scars in children with persistent UTI, as a consequence of VUR.

^99m^Tc-DTPA scintigraphy is the first method of choice for long-term monitoring of individual kidney function and structure in children with VUR and other congenital urinary tract anomalies. Additionally, it can be used as indirect radionuclide cystography, as an important independent predictor of early failure to resolve VUR.
